# Nonlinear Buckling Analysis of Functionally Graded Graphene Reinforced Composite Shallow Arches with Elastic Rotational Constraints under Uniform Radial Load

**DOI:** 10.3390/ma11060910

**Published:** 2018-05-28

**Authors:** Yonghui Huang, Zhicheng Yang, Airong Liu, Jiyang Fu

**Affiliations:** Guangzhou University-Tamkang University Joint Research Center for Engineering Structure Disaster Prevention and Control, Guangzhou University, Guangzhou 510006, China; huangyh@gzhu.edu.cn (Y.H.); cs.yeung@e.gzhu.edu.cn (Z.Y.); jiyangfu@gzhu.edu.cn (J.F.)

**Keywords:** functionally graded, graphene, elastic rotational constraints, uniform radial load, buckling, circular arch

## Abstract

The buckling behavior of functionally graded graphene platelet-reinforced composite (FG-GPLRC) shallow arches with elastic rotational constraints under uniform radial load is investigated in this paper. The nonlinear equilibrium equation of the FG-GPLRC shallow arch with elastic rotational constraints under uniform radial load is established using the Halpin-Tsai micromechanics model and the principle of virtual work, from which the critical buckling load of FG-GPLRC shallow arches with elastic rotational constraints can be obtained. This paper gives special attention to the effect of the GPL distribution pattern, weight fraction, geometric parameters, and the constraint stiffness on the buckling load. The numerical results show that all of the FG-GPLRC shallow arches with elastic rotational constraints have a higher buckling load-carrying capacity compared to the pure epoxy arch, and arches of the distribution pattern X have the highest buckling load among four distribution patterns. When the GPL weight fraction is constant, the thinner and larger GPL can provide the better reinforcing effect to the FG-GPLRC shallow arch. However, when the value of the aspect ratio is greater than 4, the flakiness ratio is greater than 103, and the effect of GPL’s dimensions on the buckling load of the FG-GPLRC shallow arch is less significant. In addition, the buckling model of FG-GPLRC shallow arch with elastic rotational constraints is changed as the GPL distribution patterns or the constraint stiffness changes. It is expected that the method and the results that are presented in this paper will be useful as a reference for the stability design of this type of arch in the future.

## 1. Introduction

Graphene-related research and application development are hot topics today, and graphene-based composites have become an important direction in application field of graphene [1,2,3,4]. Graphene and its derivatives have many advantageous material properties, such as high strength, lightweight, and high chemical stability. The tensile strength of graphene can reach 130 GPa and its theoretical elastic modulus is as high as 1 TPa [5]. As a result, graphene and its derivatives can enhance the special properties of a variety of composites. Rafiee et al. [6,7] added 0.1 wt % graphene nanoplatelets (GPLs) and carbon nanotubes (CNTs) into two composites with epoxy matrices. By comparing the mechanical properties of the two reinforced composites, they found that the GPL-reinforced composite had a higher tensile strength and fracture toughness than the CNT-reinforced composite. By thoroughly analyzing the mechanical properties of a graphene-reinforced composite with an alumina ceramic matrix, Liu et al. [8,9] found that graphene could significantly enhance the flexural strength of the composite. Based on the Mori–Tanaka micromechanical model of graphene, Ji et al. [10,11] studied the effective stiffness of graphene-reinforced materials and found that graphene could significantly enhance the effective stiffness of composites, even at a very low content. Moreover, Shen et al. [12] introduced the concept of functional grading to reinforced composites, and noted that adding a reinforcing material non-uniformly at a certain gradient significantly enhanced the properties of a composite. Using this concept, Yang et al. [13] examined the post-buckling behavior of a functionally graded multilayer graphene-reinforced nanocomposite beam. Feng et al. [14] investigated the nonlinear flexural behavior of a functionally graded graphene-reinforced composite beam. Furthering this investigation, Song and Kitipornchai et al. [15,16,17] thoroughly studied the dynamic stability of functionally graded multilayer graphene-reinforced composite beams and plates.

Due to their advantageous properties, the application of structural units that are composed of graphene-reinforced materials is of great importance in weight-sensitive engineering projects, such as aerospace engineering and long-span buildings. However, most existing studies on graphene-reinforced composite structures focus on such structural units as beams, plates, and columns; few studies have reported on graphene-reinforced arch structures. To date, only Bateni et al. [18,19] have, to some extent, investigated the nonlinear static stability of multi-functionally graded arch structures. However, they only examined arches with clamped and hinged boundaries. In practical engineering, it is difficult to realize completely clamped or completely hinged boundary constraints. The boundary constraints of arch structures have a certain rotational stiffness and are elastically constrained. Pi and Bradford et al. [20,21,22,23] performed detailed studies on the in-plane nonlinear failure of elastically constrained homogeneous circular arches and determined the critical loads that are required for them to undergo static failure under various constraint stiffness conditions. Liu [24,25,26,27,28] and Pi et al. [29,30,31] examined the out-of-plane buckling behavior of elastically constrained homogenous arches and found that the results were consistent with those of the finite element (FE) analysis.

Hence, this study examines a functionally graded graphene platelets (GPLs) reinforced composite (FG-GPLRC) shallow arch and establishes a nonlinear stable equilibrium equation for the elastically constrained FG-GPLRC shallow arch under a uniformly distributed radial load, using the Halpin-Tsai micromechanical model of graphene and the principle of virtual work. The equation is then solved to determine the critical loads of the elastically constrained FG-GPLRC shallow arch to undergo symmetric limit point buckling and antisymmetric bifurcation buckling. Finally, this study investigates the effects of the distribution pattern, mass fraction, and geometric dimensions of GPLs, as well as constraint stiffness, on the buckling behavior of elastically constrained FG-GPLRC shallow arches.

## 2. Materials Model of FG-GPLRC Shallow Arches

**Figure 1:**
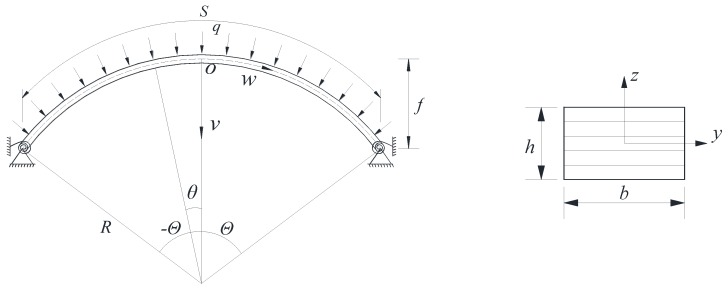
Configuration and coordinate system of the functionally graded graphene platelet-reinforced composite (FG-GPLRC) shallow arch with elastic rotational constraints.

**Figure 2:**
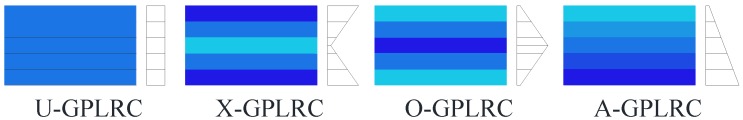
Different graphene nanoplatelets (GPL) distribution patterns in a multilayer FG-GPLRC shallow arch.

The FG-GPLRC shallow arch with elastic rotational constraints (shown in Figure 1) is composed of perfectly bonded GPLRC layers of equal thickness h_L_, which are made from a mixture of polymer matrices and GPLs. The total thickness and width of the cross-section are h and b, respectively. It is assumed that GPLs are uniformly dispersed and are randomly oriented in each layer. However, the weight fraction of GPLs varies from layer to layer. Accordingly, there are four types of GPLs distribution patterns, including U-GPLRC, X-GPLRC, O-GPLRC, and A-GPLRC, as shown in Figure 2. U-GPLRC represents that the weight fraction of GPLs in each layer is the same. X-GPLRC represents that the weight fraction of GPLs is decrease from the outer surface to the central gradually while O-GPLRC represents that the weight fraction of GPLs is increase from the outer surface to the center gradually. A-GPLRC represents that the weight fraction of GPLs is increase from the top surface to the bottom surface gradually.

The FG-GPLRC shallow arch with an even number of layers is studied in this paper. The volume fractions *V*_GPL_ of the *k*th layer for the four distribution patterns that are shown in Figure 2 are given by

(1) $$U\text{: }V_{\mathrm{GPL}}^{n}=V_{\mathrm{GPL}}^{*}$$

(2) $$X\text{: }V_{\mathrm{GPL}}^{n}=2V_{\mathrm{GPL}}^{*}\left|2k-N_{L}-1\right|/N_{L}$$

(3) $$O\text{: }V_{\mathrm{GPL}}^{n}=2V_{\mathrm{GPL}}^{*}\left(1-\left|2k-N_{L}-1\right|/N_{L}\right)$$

(4) $$A\text{: }V_{\mathrm{GPL}}^{n}=V_{\mathrm{GPL}}^{*}\left(2k-1\right)/N_{L}$$

where *N*_L_ is the total number of GPLRC layers and $V_{\mathrm{GPL}}^{*}$ is the total volume fraction of the GPLRC, which is defined as

(5) $$V_{\mathrm{GPL}}^{*}=\frac{W_{\mathrm{GPL}}}{W_{\mathrm{GPL}}+\left(\rho_{\mathrm{GPL}}/\rho_{m}\right)\left(1-W_{\mathrm{GPL}}\right)}$$

where *W*_GPL_ is the GPL weight fraction and $\rho_{\mathrm{GPL}}$ and $\rho_{m}$ are the mass densities of GPLs and polymer matrices, respectively.

The elastic modulus of composites with randomly oriented fillers can be calculated by the Halpin-Tsai model [32,33]

(6) $$E=\frac{3E_{m}\left(1-\xi_{L}\eta_{L}V_{\mathrm{GPL}}^{n}\right)}{8\left(1-\eta_{L}V_{\mathrm{GPL}}^{n}\right)}+\frac{5E_{m}\left(1+\xi_{T}\eta_{T}V_{\mathrm{GPL}}^{n}\right)}{8\left(1-\eta_{T}V_{\mathrm{GPL}}^{n}\right)}$$

in which the parameters $\eta_{L}$ and $\eta_{T}$ are defined as

(7) $$\eta_{L}=\frac{\left(E_{\mathrm{GPL}}/E_{m}\right)-1}{\left(E_{\mathrm{GPL}}/E_{m}\right)+\xi_{L}},\;\eta_{T}=\frac{\left(E_{\mathrm{GPL}}/E_{m}\right)-1}{\left(E_{\mathrm{GPL}}/E_{m}\right)+\xi_{T}}$$

and $\xi_{L}$ and $\xi_{T}$ are the filler geometry factors defined as

(8) $$\xi_{L}=2\left(a_{\mathrm{GPL}}/b_{\mathrm{GPL}}\right)\times \xi_{T}/2,\;\xi_{T}=2\left(b_{\mathrm{GPL}}/t_{\mathrm{GPL}}\right)$$

where $a_{\mathrm{GPL}}$, $b_{\mathrm{GPL}}$, and $t_{\mathrm{GPL}}$ are the length, width and thickness of GPLs, respectively. $a_{\mathrm{GPL}}/b_{\mathrm{GPL}}$, $b_{\mathrm{GPL}}/t_{\mathrm{GPL}}$ are the width-to-length ratio and width-to-thickness ratio of GPLs, respectively.

## 3. Nonlinear Equilibrium Equation

The configuration and the coordinate system of the FG-GPLRC shallow arch are shown in Figure 1. The origin o of the axis system oxy is located at the centroidal axes of the arch. The axis oz coincides with a principle axis of the cross-section, its direction changes along the circumference of the arch and it is always towards the center of the arch, and the axis ox coincides with the centroidal axis. *S*, Θ, and *R* are the axial length, half-included angle, and radius of the arch, respectively. The stiffness of the elastic rotational constraints at both ends is *k*. The longitudinal strain of an arbitrary point *Po* at the cross-section can be expressed as

(9) $$\varepsilon =\varepsilon_{m}+\varepsilon_{b}$$

where *ε_m_* is the membrane strain and *ε_b_* is the bending strain of the FG-GPLRC shallow arch. According to Donnell’s shallow arch theory [18,22], the relationship between the strain and displacement are

(10) $$\varepsilon_{m}={\tilde{w}}^{\prime }-\tilde{v}+\frac{{{\tilde{v}}^{\prime }}^{2}}{2}$$

(11) $$\varepsilon_{b}=-z\frac{{\tilde{v}}^{\prime \prime }}{R}$$

where $(\;{)}^{\prime }\equiv d(\;)/d\theta$, $\tilde{v}=v/R$ and $\tilde{w}=w/R$ are the dimensionless axial and radial displacements, respectively. *z* is the coordinate of the point *P* at the cross-section of the axis *oz*.

According to the virtual work principle, the virtual work of the FG-GPLRC shallow arch with elastic rotational constraints under uniform radial load can be expressed as

(12) $$\begin{array}{l} \delta W=\int_{-\Theta }^{\Theta }Rb\int_{-h/2}^{h/2}E\varepsilon \delta \varepsilon dzd\theta \\ \;-\int_{-\Theta }^{\Theta }qR^{2}\delta \tilde{v}d\theta +k{\tilde{v}}_{-\Theta }^{\prime }\delta {\tilde{v}}_{-\Theta }^{\prime }+k{\tilde{v}}_{\Theta }^{\prime }\delta {\tilde{v}}_{\Theta }^{\prime }=0 \end{array}$$

Substituting Equations (9)–(11) into Equation (12) leads to

(13) $$\begin{array}{l} \delta W=\int_{-\Theta }^{\Theta }\left[-NR\left(\delta {\tilde{w}}^{\prime }-\delta \tilde{v}+{\tilde{v}}^{\prime }\delta {\tilde{v}}^{\prime }\right)-M\delta {\tilde{v}}^{\prime \prime }\right]d\theta \\ \;-\int_{-\Theta }^{\Theta }qR^{2}\delta \tilde{v}d\theta +k{\tilde{v}}_{-\Theta }^{\prime }\delta {\tilde{v}}_{-\Theta }^{\prime }+k{\tilde{v}}_{\Theta }^{\prime }\delta {\tilde{v}}_{\Theta }^{\prime }=0 \end{array}$$

where *N* and *M* are the axial force and bending moment of the arch, which can be expressed as

(14) $$N=-A_{11}\left({\tilde{w}}^{\prime }-\tilde{v}+\frac{{{\tilde{v}}^{\prime }}^{2}}{2}\right)+\frac{B_{11}}{R}{\tilde{v}}^{\prime \prime }$$

(15) $$M=B_{11}\left({\tilde{w}}^{\prime }-\tilde{v}+\frac{\tilde{v}}{2}\right)-\frac{D_{11}}{R}{\tilde{v}}^{\prime \prime }$$

in which, *A*_11_, *B*_11_, and *D*_11_ are the stiffness components of the FG-GPLRC shallow arch, which are defined as

(16) $$\left\{A_{11}\;B_{11}\;D_{11}\right\}=b\sum_{n=1}^{N_{L}}\int_{z_{n}}^{z_{n+1}}E^{\left(n\right)}\left\{1\;z\;z^{2}\right\}\;\mathrm{dz}$$

where *z_n_* and *z_n_*_+1_ are the coordinates of the bottom and upper surfaces of the *k*th GPLRC layer, respectively.

Integrating Equation (13) by parts leads

(17) $$\begin{array}{l} \int_{-\Theta }^{\Theta }\left\{{\left(NR\right)}^{\prime }\delta \tilde{w}+\left[NR+{\left(NR\tilde{v}\right)}^{\prime }-M^{\prime \prime }-qR^{2}\right]\delta \tilde{v}\right\}d\theta \\ -{\left[NR\delta \tilde{w}+\left(NR{\tilde{v}}^{\prime }-M^{\prime }\right)\delta \tilde{v}\right]}_{-\Theta }^{\Theta }+{\left[\left(-M\pm k{\tilde{v}}^{\prime }\right)\delta {\tilde{v}}^{\prime }\right]}_{-\Theta }^{\Theta }=0 \end{array}$$

At the same time, the following essential kinematic boundary conditions

(18) $$\begin{array}{ll} \delta \tilde{v}=0\;\mathrm{and}\;\delta \tilde{w}=0 & \mathrm{at}\;\theta =\text{±}\Theta \\ -M\text{ ± }k{\tilde{v\;}}^{\prime }=0 & \mathrm{at}\;\theta =\text{±}\Theta \end{array}$$

need to be satisfied. Hence, the last two terms of Equation (17) vanish.

Because $\delta \tilde{v}=0$ and $\delta \tilde{w\;}=0$ are arbitrary by definition, to hold Equation (17), the differential equations of equilibrium have to be satisfied

(19) $${\left(NR\right)}^{\prime }=0$$

(20) $$NR+{\left(NR{\tilde{v}}^{\prime }\right)}^{\prime }-M^{\prime \prime }-qR^{2}=0$$

The essential kinematic boundary conditions are

(21) $$\begin{array}{l} \tilde{v}\left(\Theta \right)=\tilde{v}\left(-\Theta \right)=\tilde{w}\left(\Theta \right)=\tilde{w}\left(-\Theta \right)=0\\ -M\left(\Theta \right)+k{{\tilde{v}}^{\prime }}_{\Theta }=-M\left(\Theta \right)-k{{\tilde{v}}^{\prime }}_{-\Theta }=0 \end{array}$$

Substituting Equation (14) into Equation (15) leads to the bending moment of the FG-GPLRC elastic rotational constraint arch, as

(22) $$M=-\left(D_{11}-\frac{B_{11}^{2}}{A_{11}}\right)\frac{{\tilde{v}}^{\prime \prime }}{R}-\frac{B_{11}}{A_{11}}N$$

Substituting Equations (19) and (22) into Equation (20), the differential equation of equilibrium in the radial direction can be rewritten as

(23) $$\frac{{\tilde{v}}^{iv}}{\mu^{2}}+{\tilde{v}}^{\prime \prime }=P$$

where, *P* is a dimensionless load, which is defined as

(24) $$P=\frac{qR-N}{N}$$

*μ* is dimensionless axial force parameter, which is defined as

(25) $$\mu^{2}=\frac{NR^{2}}{\kappa },\;\kappa =D_{11}-\frac{B_{11}^{2}}{A_{11}}$$

where κ is the equivalent stiffness of the FG-GPLRC shallow arch.

The dimensionless radial displacement $\tilde{v}$ can be obtained by solving Equation (23) under the boundary conditions given by Equation (21)

(26) $$\tilde{v}=\frac{P}{\mu^{2}}\left[\frac{\cos\left(\mu \theta \right)-\cos\beta }{\cos\beta }\Gamma +\frac{\mu^{2}\theta^{2}-\beta^{2}}{2}\right]+\frac{\cos\left(\mu \theta \right)-\cos\beta }{\left(1+2\alpha \right)\cos\beta }\frac{2\alpha B_{11}\Gamma }{A_{11}R}$$

in which, β=μΘ and *α* is the ratio of the bending stiffness per unit length of the equivalent stiffness κ/S to the stiffness of elastic rotational end constraints *k*, which can be expressed as

(27) $$\alpha =\frac{\kappa }{kS}$$

Substituting Equations (25) and (26) into (14), and integrating the function over the entire arch, leads to the nonlinear equilibrium equation between the axial force parameter *μ* and the dimensionless load *P*, expressed as

(28) $$A_{1}P^{2}+B_{1}P+C_{1}=0$$

where

(29) $$A_{1}=\frac{1}{4\beta^{2}}\left(\frac{\tan\beta }{\beta }-\frac{1}{\cos^{2}\beta }\right)\Gamma^{2}-\frac{1}{\beta^{2}}\left(1-\frac{\tan\beta }{\beta }\right)\Gamma -\frac{1}{6}$$

(30) $$B_{1}=\frac{1}{\beta^{2}}\left(1-\frac{\tan\beta }{\beta }\right)\Gamma +\frac{1}{3}-\frac{\alpha \sin2\beta +2\alpha \beta }{2\beta \left(1+2\alpha \right)\cos^{2}\beta }\frac{B\Gamma^{2}}{\lambda h}+\frac{2\alpha \beta -\tan\beta -4\alpha \tan\beta }{\beta \left(1+2\alpha \right)}\frac{B\Gamma }{\lambda h}+\frac{B}{\lambda h}$$

(31) $$C_{1}=\left[\frac{\alpha^{2}\beta \sin2\beta -2\alpha^{2}\beta^{2}}{2\cos^{2}\beta {\left(1+2\alpha \right)}^{2}}-\frac{2\alpha \beta \tan\beta }{\Gamma \left(1+2\alpha \right)}\right]{\left(\frac{B\Gamma }{\lambda h}\right)}^{2}+\frac{2\alpha \left(\tan\beta -\beta \right)}{\beta \left(1+2\alpha \right)}\frac{B\Gamma }{\lambda h}-\frac{\beta^{2}\kappa }{A_{11}\lambda^{2}h^{2}}$$

in which

(32) $$\Gamma =\frac{1+2\alpha }{2\alpha +\tan\beta /\beta }$$

*λ* is the geometric parameter of FG-GPLRC shallow arch defined as

(33) $$\lambda =\frac{R\Theta^{2}}{h}=\frac{S\Theta }{2h}$$

which increases as the arch length and the half-included angle increase but decreases as the thickness of cross section increases.

Thus, Equation (28) establishes the relationship between the external load *P* and the axial compressive force parameter *β*. By solving Equation (28), the axial compressive force *β* under a different load *P* can be obtained. Then, by substituting *β* into Equation (26), the dimensionless radial displacement $\tilde{v}$ can be obtained. Thus, the *P*-$\tilde{v}$ curve can be drawn, and the nonlinear buckling behavior can be analyzed.

## 4. Buckling Analysis

### 4.1. Limit Instability Buckling

The limit instability points are local maximum and minimum points on the nonlinear equilibrium path of an FG-GPLRC shallow arch, which can be determined by routing calculus. The localized load *P* can be expressed as an implicit function of the parameter *β* using Equation (28) as *F*(*P*, *β*) = 0. Hence, the limit loads can be obtained by setting

(34) $$\frac{dP}{d\beta }=-\frac{\partial F\left(P,\beta \right)/\partial \beta }{\partial F\left(P,\beta \right)/\partial P}=0$$

which leads to the equation of equilibrium between the dimensionless load *P* and the axial force parameter *β* at the limit points, which is expressed as

(35) $$A_{2}P^{2}+B_{2}P+C_{2}=0$$

where

(36) $$A_{2}=\frac{\beta \partial A_{1}}{\partial \beta }-4A_{1},B_{2}=-4A_{1}+\frac{\beta \partial B_{1}}{\partial \beta }-2B_{1},C_{2}=-2B_{1}+\frac{\beta \partial C_{1}}{\partial \beta }$$

Solving Equations (28) and (35) simultaneously can yield the axial force parameter *β* and the limit point instability load *P*. Then, by substituting *β* and *P* into Equation (26), the dimensionless radial displacement $\tilde{v}$, corresponding to the limit instability loads *P* can be obtained.

### 4.2. Bifurcation Buckling

The FG-GPLRC shallow arches with elastic rotational constraints may also buckle in a bifurcation mode. The critical condition for buckling may be stated that the second variation of the total potential of the arch and load system is equal to zero for any admissible infinitesimal deformation variations. Thus, the differential equation for bifurcation buckling is [22].

(37) $$\frac{{\tilde{v}}^{iv}}{\mu^{2}}+{\tilde{v}}^{\prime \prime }=0$$

The general solution of Equation (37) is

(38) $${\tilde{v}}_{b}=E_{1}+E_{2}\theta +E_{3}\sin\left(\mu \theta \right)+E_{4}\cos\left(\mu \theta \right)$$

where *E*_1_, *E*_2_, *E*_3_, and *E*_4_ are the undetermined coefficients.

The boundary conditions for deformations are

(39) $$\{\begin{array}{l} {\tilde{v}}_{b}={\tilde{w}}_{b}=0\\ \frac{\kappa {{\tilde{v}}^{\prime \prime }}_{b}}{R}\pm k{{\tilde{v}}^{\prime }}_{b}=0 \end{array},\;\theta =\pm \Theta$$

Substituting Equation (38) into Equation (39) obtains

(40) $$\left[\begin{array}{cccc} 1 & -\Theta & -\sin\beta & \cos\beta \\ 1 & \Theta & \sin\beta & \cos\beta \\ 0 & -1 & 2\mu^{2}\Theta \alpha \sin\beta -\mu \cos\beta & -2\mu^{2}\Theta \alpha \cos\beta -\mu \sin\beta \\ 0 & 1 & -2\mu^{2}\Theta \alpha \sin\beta +\mu \cos\beta & -2\mu^{2}\Theta \alpha \cos\beta -\mu \sin\beta \end{array}\right]\left\{\begin{array}{c} E_{1}\\ E_{2}\\ E_{3}\\ E_{4} \end{array}\right\}=\left\{\begin{array}{c} 0\\ 0\\ 0\\ 0 \end{array}\right\}$$

The existence of a nontrivial solution to Equation (40) for *E*_1_–*E*_4_ requires that their determinant of the coefficient matrix vanishes, which leads to the characteristic equation:

(41) $$\left[\frac{\sin\beta }{2\alpha }+\beta^{2}\left(\sin\beta -\frac{\cos\beta }{2\alpha \beta }\right)\right]\left(\cos\beta +\frac{\sin\beta }{2\alpha \beta }\right)=0$$

When the first term of Equation (41) is equal to zero, there is

(42) $$\tan\beta =\frac{\beta }{1+2\alpha \beta^{2}}$$

Solving Equation (42) leads to

(43) $$\beta =\mu_{b}\Theta =\eta_{b}\pi$$

Substituting Equation (43) into Equation (25), the axial compressive force *N_b_* of the FG-GPLRC shallow arch with elastic rotational constraints corresponding to an antisymmetric buckling is obtained as 

(44) $$N_{b}=\frac{\mu_{b}^{2}\kappa }{R^{2}}=\frac{{\left(\eta_{b}\pi \right)}^{2}\kappa }{{\left(S/2\right)}^{2}}$$

Substituting Equation (43) into Equation (28), the critical load *P_b_* of the FG-GPLRC shallow arch with elastic rotational constraints corresponding to an antisymmetric buckling is obtained, as 

(45) $$P_{b}=\frac{-B_{b}\pm \sqrt{B_{b}^{2}-4A_{b}C_{b}}}{2A_{b}}$$

The existence of real solutions for *P_b_* require that expressions in the square root of Equation (45) is non-negative, i.e.,

(46) $$B_{b}^{2}-4A_{b}C_{b}\geq 0$$

From Equations (29)–(31), it can be seen that, when $\beta =\eta_{b}\pi$ and the GPL distribution model is given, the Equation (45) is a function of *λ* and *α*. By solving Equation (46), the minimum geometric parameter $\lambda_{b}$ of the FG-GPLRC shallow arch, at which antisymmetric buckling is possible can be solved.

When the second factor of Equation (41) is equal to zero, there is

(47) tanβ=−2αβ

From Equation (47), the solution for *β* can be obtained as

(48) $$\beta =\mu_{s}\Theta =\eta_{s}\pi$$

which is a parameter related to the value of α. Substituting Equation (48) into Equation (25), the axial compressive force *N_s_* of the FG-GPLRC shallow arch with elastic rotational constraints is obtained as

(49) $$N_{s}=\frac{{\left(\eta_{s}\pi \right)}^{2}\kappa }{{\left(S/2\right)}^{2}}$$

Substituting the solution given by Equation (47) into Equation (28) leads to

(50) P=0

By solving Equations (24), (49) and (50), the lowest buckling load *qR* can be obtained as

(51) $$qR=N_{s}=\frac{{\left(\eta_{s}\pi \right)}^{2}\kappa }{{\left(S/2\right)}^{2}}$$

which indicates that the normal axial compressive force *qR* during buckling is equal to the axial compressive force *N_s_*.

## 5. Comparison and Discussion

In order to meet the requirement of Donnell’s shallow shell theory, in the following numerical FG-GPLRC shallow arch models, the width of section is chosen as *b* = 0.3 m, the total thickness is chosen as *h* = 0.25 m, and the axial length of the arch is chosen as *S* = 20 m. The geometric parameters of GPL are $a_{\mathrm{GPL}}$ = 2.5 µm, $b_{\mathrm{GPL}}$ = 1.5 µm and $t_{\mathrm{GPL}}$ = 1.5 nm. Young’s modulus of polymer matrix and GPL are 3 GPa and 1010 GPa, respectively. The density of the polymer matrix and GPL are 1200 kg/m^3^ and 1062.5 kg/m^3^, respectively. The material properties of GPLs that are used in this paper are referred from Ref. [6].

### 5.1. Effect of the Number of Nanocomposite Layers on the Numerical Results

When a multilayer graphene-reinforced nanocomposite is used to simulate functionally graded properties, the number of layers of the nanocomposite (*N*_L_) will significantly affect the simulation results. Hence, the elastically constrained FG-GPLRC shallow arch was simulated using GPLRCs with varying numbers of layers to examine the effect of the number of nanocomposite layers on the critical buckling load of the arch. The calculated results are summarized in Table 1. Note that the limit point buckling loads of FG-GPLRC shallow arches are obtained by solving Equations (28) and (35) simultaneously, while the bifurcation loads are obtained by solving Equation (45).

As demonstrated in Table 1, there is a relatively insignificant difference between the buckling loads of the elastically constrained functionally graded X-GPLRC arch, with *N*_L_ = 20 and *N*_L_ = 1000. This suggests that the layers number of the nanocomposite *N*_L_ = 20 is sufficient to facilitate an accurate simulation of functionally graded property, whose thickness is continuously changing. Therefore, *N*_L_ = 20 was used in the subsequent numerical simulation and analysis.

**Figure 3:**
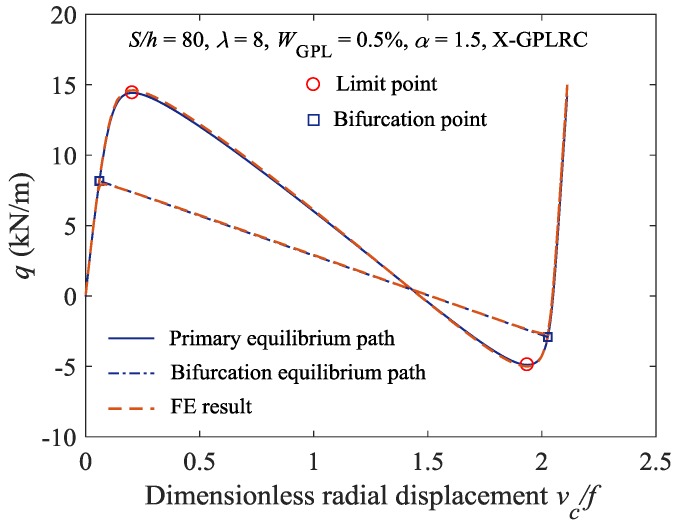
Comparison of analytical results and finite element (FE) results.

Additionally, the proposed analytical solutions of the stable equilibrium path and the buckling load of the elastically constrained FG-GPLRC shallow arch are validated by finite element (FE) solutions, which were obtained using the commercial FE software ANSYS. The FG-GPLRC shallow arch model was constructed using SHELL181 layered elements and the elastic constraints at the ends were established using COMBIN14 elements. The circular arch was meshed into 100 SHELL181 elements and six COMBIN14 elements. A comparison of the analytical and FE solutions is shown in Figure 3.

In Figure 3, *v*_c_ and *f* are the radial displacement and rise of the FG-GPLRC shallow arch. The analytical solutions are highly consistent with the FE simulation results, which indicates that the analytical method proposed in this study can be used to obtain a sufficiently accurate stable equilibrium path and critical buckling load of the elastically constrained FG-GPLRC shallow arch.

### 5.2. Limit Point Buckling Analysis

The limit point buckling loads of the elastically constrained arch when reinforced with GPLs in various distribution patterns are compared in Table 2. GPLs of various distribution patterns (U-, X-, O-, and A-types) significantly increase the critical buckling load of the elastically constrained pure epoxy resin arch, as demonstrated in Table 2. In addition, GPLs of the X-type distribution pattern increase the critical buckling load of the elastically constrained pure epoxy resin arch to the highest extent, followed by GPLs of the U-, A-, and O-type distribution patterns, in that order. This is because the elastically constrained composite arch reinforced with GPLs of the X-type distribution pattern contains a relatively large amount of reinforcing GPL filler in the top and bottom layers and is capable of resisting a relatively high bending stress; consequently, the critical buckling load of this type of reinforced composite arch is relatively high. GPLs with the O-type distribution pattern, the opposite of the X-type distribution pattern, increase the critical buckling load of the elastically constrained composite arch relatively insignificant. This is because most of the GPL fillers in the O-type distribution are concentrated near the neutral axis of the section, leading to a minor increase in cross sectional bending stiffness.

**Figure 4:**
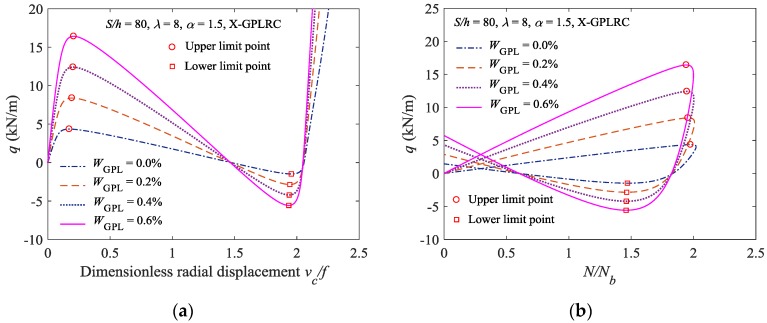
Effect of the GPLs weight fraction on the limit point buckling load: (**a**) Load displacement curve; and; (**b**) load-axial force curve.

Figure 4 shows the nonlinear equilibrium paths and buckling loads for the elastically constrained FG-GPLRC shallow arch with various mass fractions of GPLs in the X-type distribution pattern (*W*_GPL_ = 0.0% signifies the pure epoxy resin arch). As shown in Figure 4, as the mass fraction of GPLs increases, the critical load for the elastically constrained FG-GPLRC shallow arch to undergo limit point buckling increases, and the stability of the elastically constrained FG-GPLRC shallow arch increases. In addition, when compared to the elastically constrained pure epoxy resin arch, the enhancing effects of GPLs increase significantly as the GPL content increases.

**Figure 5:**
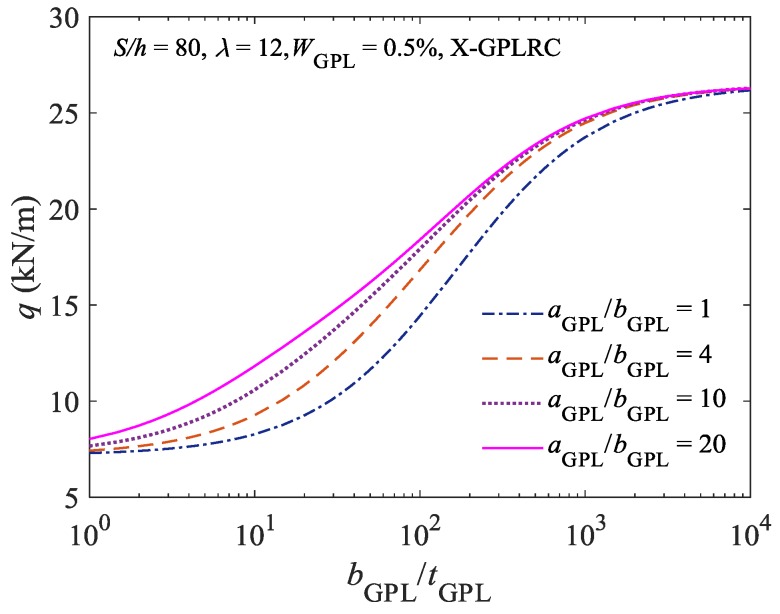
Effect of GPLs geometric dimensions on the limit point buckling load.

Figure 5 shows the effects of the geometric dimensions of GPLs on the limit point buckling load of the elastically constrained X-type FG-GPLRC shallow arch when the GPL content is fixed. As demonstrated in Figure 5, when the height/width (a_GPL_/b_GPL_) ratio of GPLs is fixed, the buckling load for the elastically constrained X-type FG-GPLRC shallow arch increases as the width/thickness (b_GPL_/t_GPL_) ratio increases. When the b_GPL_/t_GPL_ ratio is greater than $10^{3}$, the effect of the b_GPL_/t_GPL_ ratio on the buckling load is no longer significant. When the b_GPL_/t_GPL_ ratio is fixed, the buckling load increases as the a_GPL_/b_GPL_ ratio increases. However, when the a_GPL_/b_GPL_ ratio is greater than 4, the effect of the a_GPL_/b_GPL_ ratio on the buckling load gradually decreases as the a_GPL_/b_GPL_ ratio increases. Specifically, the greater the a_GPL_/b_GPL_ and b_GPL_/t_GPL_ ratios of GPLs, the larger the surface area of GPLs, and the smaller the thickness of GPLs. Therefore, within a certain range, the larger and thinner GPLs have more significant enhancing effect on the stable bearing capacity of the elastically constrained composite shallow arch.

**Figure 6:**
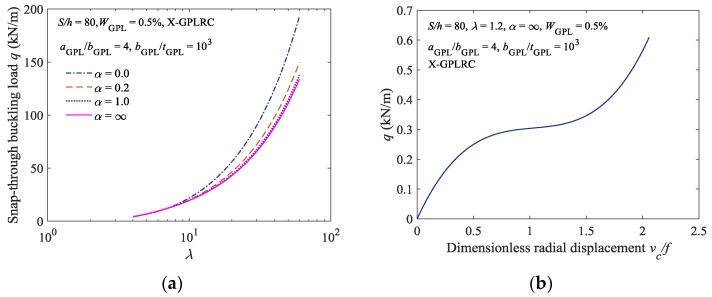
Effect of constraint stiffness and geometry of arches on the limit point buckling load: (**a**) Limit point buckling load; and; (**b**) load displacement curve (*λ* = 1.5).

Figure 6a shows the limit point buckling loads for the elastically constrained X-type FG-GPLRC shallow arch under various constraint stiffness conditions. The geometric parameter *λ* of the elastically constrained FG-GPLRC shallow arch varies between 4 and 60. Specifically, the constraint stiffness parameters α=0 and α=∞ signify a completely clamped FG-GPLRC shallow arch and a completely hinged FG-GPLRC shallow arch, respectively. As shown in Figure 6a, as *α* gradually increases from 0 to ∞, the FG-GPLRC shallow arch gradually transitions from being a completely clamped arch to being a completely hinged arch, and the buckling load of the arch gradually decreases. When *α* = 1, the critical buckling load of the elastically constrained FG-GPLRC shallow arch is very close to that of a completely hinged FG-GPLRC shallow arch, thus suggesting that a certain low stiffness can facilitate a relatively satisfactory simulation of a completely hinged FG-GPLRC shallow arch.

In addition, the limit point buckling load of the elastically constrained X-type FG-GPLRC shallow arch increases as the geometric parameter λ increases. When λ is relatively low, the elastically constrained FG-GPLRC shallow arch is equivalent to a curved beam that undergoes nonlinear bending under loading; it has no extreme points for limit point buckling, and thus will not undergo buckling failure, as shown in Figure 6b.

### 5.3. Bifurcation Buckling Analysis

According to Equation (46), when the elastically constrained FG-GPLRC shallow arch undergoes antisymmetric bifurcation buckling, its geometric parameter must meet the following condition: $\lambda \geq \lambda_{b}$. The values of the critical geometric parameter $\lambda_{b}$ required for the arch to undergo bifurcation buckling of the FG-GPLRC arches under a *α* = 1.5 elastic constraint condition are summarized Table 3.

As demonstrated in Table 3, when α is fixed, the value of $\lambda_{b}$ for the O-type FG-GPLRC shallow arch to undergo bifurcation buckling is the smallest, suggesting that the elastically constrained FG-GPLRC shallow arch with GPLs in the O-type distribution pattern is the most prone to bifurcation buckling, followed by the FG-GPLRC shallow arches with GPLs of the U-, A-, and X-type distribution patterns, in that order.

**Figure 7:**
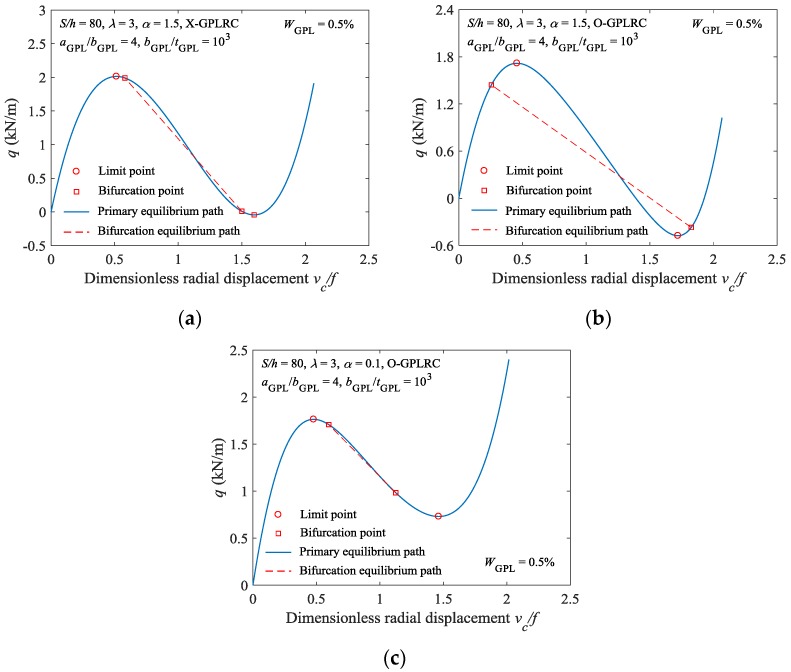
Effect of constraint stiffness and GPLs distribution patterns on buckling model: (**a**) Load displacement curve (X-GPLRC, *α* = 1.5); (**b**) load displacement curve (O-GPLRC, *α* = 1.5); and, (**c**) load displacement curve (O-GPLRC, *α* = 0.1).

Figure 7 shows the buckling behavior of the FG-GPLRC shallow arch with GPLs in various distribution patterns under various constraint stiffness conditions. As shown in Figure 7a, when α=1.5, the bifurcation buckling point of the X-type FG-GPLRC shallow arch occurs after its limit point buckling point, indicating that limit point buckling will occur before bifurcation buckling for this type of arch. Under the same condition, the bifurcation buckling point of the O-type FG-GPLRC shallow arch occurs before its limit point buckling point, indicating that bifurcation buckling will occur before limit point buckling for this type arch (Figure 7b). This suggests that the distribution pattern of GPLs can alter the buckling mode of elastically constrained FG-GPLRC shallow arches. In addition, the stable equilibrium path for the O-type FG-GPLRC shallow arch when α=0.1 was analysed, as shown in Figure 7c. A comparison of Figure 7b,c shows that, under the same conditions, altering the constraint stiffness can result in a change in the buckling mode of the FG-GPLRC shallow arch.

**Figure 8:**
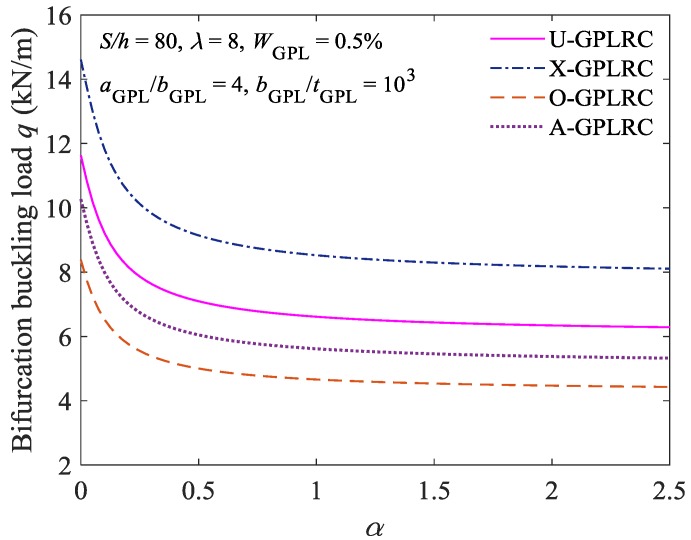
Effect of constraint stiffness on bifurcation buckling load.

Figure 8 shows the critical bifurcation buckling loads for the FG-GPLRC shallow arch under various constraint stiffness conditions (i.e., various values of *α*). As demonstrated in Figure 8, as *α* gradually increases, the constraint at the ends of the FG-GPLRC shallow arch gradually transitions from being a clamp constraint to being a hinge constraint, and the critical bifurcation buckling load gradually decreases. In addition, for the FG-GPLRC shallow arch with GPLs of various distribution patterns, the critical bifurcation buckling load of the FG-GPLRC shallow arch with GPLs of the X-type distribution pattern is the largest, followed by those of the FG-GPLRC shallow arch with GPLs of the U-, A-, and O-type distribution patterns, in that order. This finding is consistent with the conclusion that is derived from Table 2.

## 6. Conclusions

To investigate the buckling behavior of an elastically constrained FG-GPLRC shallow arch under uniform radial load, this study establishes the nonlinear stable equilibrium equation for this type of arch while using the Halpin-Tsai micromechanical model of graphene and the principle of virtual work. The equation is then solved to determine the critical load of the elastically constrained FG-GPLRC shallow arch to undergo symmetric limit point buckling and antisymmetric bifurcation buckling under uniform radial load. Next, the proposed method that is employed in this study is validated through a finite element analysis. A parametric analysis was also carried out using the proposed theoretical method, and the results show that GPLs in various distribution patterns (U-, X-, O-, and A-types) all increase to some extent the critical buckling load of the elastically constrained pure epoxy resin arch. The critical buckling load of the elastically FG-GPLRC shallow arch reinforced with GPLs of the X-type distribution pattern, is the highest, so this type arch is the most stable. When the total graphene content is fixed, the enhancing effects increase as the a_GPL_/b_GPL_ and b_GPL_/t_GPL_ ratios of graphene increase. When the a_GPL_/b_GPL_ ratio is greater than 4 and the b_GPL_/t_GPL_ ratio is greater than $10^{3}$, the enhancing effects of the geometric shape of graphene on the buckling load of the arch are no longer significant. As *α* gradually increases, the constraint at the ends of the FG-GPLRC shallow arch gradually transitions from a clamp constraint to a hinge constraint, and the critical buckling load decreases. Altering the distribution pattern of graphene or the stiffness of the constraint at the ends of the FG-GPLRC shallow arch can result in a change in the buckling mode of the arch. Functionally graded graphene-reinforced composites have advantageous material properties, and one can therefore expect that it will be more and more commonly applied in engineering as the development of material manufacturing technology. The method and results that are presented in this paper would be useful as a reference for the stability design of the FG-GPLRC shallow arches in the future.

## Figures and Tables

**Table 1:** Effect of layers number *N*_L_ on critical buckling load (X-GPLRC, *α* = 1.5, *S*/*h* = 80, *W*_GPL_ = 0.5%, *λ* = 8).

*N*_L_	Limit Point Buckling	Bifurcation Buckling
4	13.823	7.776
6	14.218	8.138
10	14.419	8.175
20	14.503	8.228
1000	14.531	8.247

**Table 2:** Effect of graphene nanoplatelets (GPL) distribution patterns on buckling load (*S*/*h* = 80, W_GPL_ = 0.5%, *λ* = 12).

*α*	U	X	O	A	Pure
0	22.836	28.202	16.941	20.406	8.584
1.5	18.952	24.110	13.580	16.873	7.124
∞	18.764	23.913	13.418	16.158	7.053

**Table 3:** Critical geometrical parameter *λ_b_* (*S*/*h* = 80, a_GPL_/b_GPL_ = 4, b_GPL_/t_GPL_ = 10^3^, *W*_GPL_ = 0.5%).

Patterns	U	X	O	A
*λ_b_*	2.3584	2.6912	1.9702	2.4167
